# In Vitro Evaluation of CD276-CAR NK-92 Functionality, Migration and Invasion Potential in the Presence of Immune Inhibitory Factors of the Tumor Microenvironment

**DOI:** 10.3390/cells10051020

**Published:** 2021-04-26

**Authors:** Stefan Grote, Guillermo Ureña-Bailén, Kenneth Chun-Ho Chan, Caroline Baden, Markus Mezger, Rupert Handgretinger, Sabine Schleicher

**Affiliations:** Department of Hematology and Oncology, Children’s Hospital, University Hospital Tuebingen, 72076 Tuebingen, Germany; guillermo.urena@med.uni-tuebingen.de (G.U.-B.); kc220@outlook.com (K.C.-H.C.); caroline.baden@student.uni-tuebingen.de (C.B.); markus.mezger@med.uni-tuebingen.de (M.M.); rupert.handgretinger@med.uni-tuebingen.de (R.H.); sabine.schleicher@med.uni-tuebingen.de (S.S.)

**Keywords:** CD276, B7-H3, chimeric antigen receptor, NK-92, immune therapy, melanoma, CRISPR/Cas9

## Abstract

Background: Melanoma is the most lethal of all skin-related cancers with incidences continuously rising. Novel therapeutic approaches are urgently needed, especially for the treatment of metastasizing or therapy-resistant melanoma. CAR-modified immune cells have shown excellent results in treating hematological malignancies and might represent a new treatment strategy for refractory melanoma. However, solid tumors pose some obstacles for cellular immunotherapy, including the identification of tumor-specific target antigens, insufficient homing and infiltration of immune cells as well as immune cell dysfunction in the immunosuppressive tumor microenvironment (TME). Methods: In order to investigate whether CAR NK cell-based immunotherapy can overcome the obstacles posed by the TME in melanoma, we generated CAR NK-92 cells targeting CD276 (B7-H3) which is abundantly expressed in solid tumors, including melanoma, and tested their effectivity in vitro in the presence of low pH, hypoxia and other known factors of the TME influencing anti-tumor responses. Moreover, the CRISPR/Cas9-induced disruption of the inhibitory receptor NKG2A was assessed for its potential enhancement of NK-92-mediated anti-tumor activity. Results: CD276-CAR NK-92 cells induced specific cytolysis of melanoma cell lines while being able to overcome a variety of the immunosuppressive effects normally exerted by the TME. NKG2A knock-out did not further improve CAR NK-92 cell-mediated cytotoxicity. Conclusions: The strong cytotoxic effect of a CD276-specific CAR in combination with an “off-the-shelf” NK-92 cell line not being impaired by some of the most prominent negative factors of the TME make CD276-CAR NK-92 cells a promising cellular product for the treatment of melanoma and beyond.

## 1. Introduction

Malignant cutaneous melanoma, which develops from the pigment forming cells of the skin, is a highly aggressive tumor characterized by an increasing worldwide incidence [1]. Although melanoma accounts for only 4% of all skin cancers, it causes the highest number of skin cancer-related deaths [2]. The majority of patients diagnosed with melanoma have early-stage disease, and the prognosis is generally favorable although heterogeneous. In contrast, outcome for patients with metastatic disease is poor, with a five-year survival rate of approximately 10% [3].

Besides targeted therapy with BRAF and MEK inhibitors in BRAF V600 mutated melanomas, immune checkpoint inhibitors have revolutionized the treatment of patients with advanced melanoma and other cancers. Blockade of the CTLA-4 or/and PD-1 signaling axis enhances T-cell-mediated antitumor immune responses, leading to improved survival and durable responses in patients [4]. However, a significant proportion of patients either does not respond or show progression after the initial response to checkpoint inhibitor treatment. Multiple mechanisms, such as downregulation of the immune checkpoint ligands by the tumor, activation of alternative cancer signaling pathways, mutations in genes involved in IFN-γ signaling, immuno-editing and changes in the tumor microenvironment (TME) have been shown to contribute to both primary and acquired resistance to immune checkpoint inhibitors [5,6]. A further challenge is posed by their mode of action, as immune checkpoint inhibitors can also induce immune-related adverse effects that require careful monitoring and immediate treatment [7,8,9]. Therefore, new treatment strategies for therapy-resistant progressed melanoma are still of great significance.

Novel immunotherapeutic approaches aim at utilizing the innate cytotoxic ability of lymphocytes by specifically redirecting immune cells to target tumor cells using chimeric antigen receptors (CAR), synthetic recombinant receptors that combine target antigen binding with immune cell activation [10]. Most prominently, CD19-targeting autologous T cells were recently approved by the US Food and Drug Administration (FDA) as well as the European Medicines Agency (EMA) for the treatment of relapsed or refractory B-cell malignancies. CD19-CAR T cells have demonstrated tremendous clinical responses in treating acute lymphoblastic leukemia and certain types of relapsed or refractory large B cell lymphoma resulting in preponed approval after evaluation of phase 2 clinical studies [11,12]. The occurrence of, partially, severe side effects such as cytokine release syndrome (CRS) or neurotoxic events as well as the extensive manufacturing process of a custom, autologous T cell product, have yet hindered the inclusion of CAR T cells as a general treatment option [13].

NK cells have become an increasingly attractive alternative effector cell source for CAR-modification because of their potent innate anti-tumor activity. Phase 1 and 2 trials with CD19-CAR-engineered NK cells have shown impressive responses in patients with relapsed or refractory non-Hodgkin’s lymphoma (NHL) or chronic lymphocytic leukemia (CLL). In addition, associated side effects such as graft-versus-host disease (GvHD), CRS or severe neurotoxicity were not reported [14]. However, the majority of studies including CAR-modified NK cells so far have been performed with NK-92 cells. NK-92 is an IL-2-dependent, continuously expanding human NK cell line which exhibits phenotypic and functional characteristics of activated, primary NK cells that has been approved by the FDA for patient treatment [15]. NK-92 cells were shown to be easily expandable in a GMP-compliant manner to sufficient cell numbers for clinical use [16]. Moreover, irradiated NK-92 cells have demonstrated clinical safety and persistent anti-tumor activity against hematological malignancies as well as solid cancers [17,18,19,20,21]. Compared to primary NK cells, they only express few inhibitory receptors such as NKG2A [22]. CAR-modified NK-92 cells could be manufactured as an “off-the-shelf”-available CAR immune cell product, reducing production complexity and, subsequently, financial expenditure [23]. Several early phase clinical trials with CAR-engineered NK-92 cells are currently being carried out in Europe, China and the US (clinicaltrials.gov; NCT 02742727, 02839954, 02892695, 03656705, 03940833).

Along with the tremendous success CAR therapies have had in treating hematological malignancies, so far, they have only demonstrated limited effectivity against solid tumors [24]. The biggest challenges are the lack of tumor-specific antigens, tumor antigen heterogeneity, immune cell homing and infiltration as well as the immunosuppressive TME.

CD276 (B7-H3) is a member of the B7 superfamily of immune checkpoints and recently emerged as an important prognostic tumor marker as well as a promising target structure for immunotherapy [25,26,27]. Although CD276 mRNA is reportedly ubiquitously expressed in various human tissue types, assessment of CD276 translational status using immunohistochemistry demonstrated only weak or absent protein expression on normal tissue [28,29]. Importantly, CD276 is overexpressed in a variety of solid tumors, including melanoma [27]. Such overexpression was reported to play a pivotal role in tumor vascularization, metastasis and overall poor clinical prognosis [30,31]. Since CD276 is expressed on different cell types within the tumor, like differentiated as well as stem-like cells or even tumor vasculature, immunotherapy targeting B7-H3 may have great potential for complete tumor eradication [32,33]. So far, different anti-CD276 blocking antibodies have been examined in pre-clinical as well as phase 1 clinical studies and have demonstrated a good safety and powerful anti-tumor profile [34,35]. Additionally, CD276-specific chimeric antigen receptors have been developed and tested as CAR-engineered T cells in multiple pre-clinical studies targeting various solid tumors including melanoma [36,37,38,39,40].

The tumor microenvironment consists of a variety of cells including tumor-associated fibroblasts, macrophages, dendritic cells, neutrophils, regulatory T cells (Tregs) and myeloid derived suppressor cells (MDSC) as well as extracellular matrix and multiple secreted or cell membrane-presented molecules. Due to the immunosuppressive properties of the TME, tumor cells are able to escape immune-surveillance by impairing immune cell infiltration and cytotoxic functions, thus supporting tumor growth and metastasis [41].

To elicit a strong anti-tumor immune response in solid tumors, CAR-engineered NK cells need to overcome major challenges, such as insufficient homing and infiltration due to the lack of cognate chemokine signals and physiological barriers, immunosuppressive cells and cytokines, low pH, oxidative stress and lack of immune-stimulating cytokines [42,43]. Soluble factors such as TGFβ, which are highly secreted within the TME, have been shown to facilitate metastatic development as well as tumor escape mechanisms during treatment with primary immune cells [44,45]. TGFβ directly impairs NK cell immune effector function through inhibition of the T-bet transcription factor (SMAD3) which reduces cytotoxicity and IFN-γ secretion [42,46]. Furthermore, TGFβ upregulates expression of NK cell inhibitory receptors while at the same time downregulating activating receptors such as NKp30 and NKG2D or their respective ligands, such as MHC class I polypeptide-related sequence A (MICA), on cancer cells [47,48]. Moreover, metabolic dysregulation by the induction of nutrient-catabolizing enzymes such as indoleamine 2,3-dioxygenase (IDO) and the secretion of large amounts of lactate promote an acidic tumor environment and is linked to growth arrest of tumor-infiltrating NK cells [49,50]. Lactate accumulation in the TME is associated with limited NK cell-mediated cytotoxicity through downregulation of activating NK receptors [51]. Furthermore, melanoma cells exposed to an acidic pH demonstrate elevated levels of invasiveness and metastatic development [52,53]. Another factor impairing effectivity of tumor-infiltrating NK cells is the hypoxic environment within solid tumors. Hypoxia has been shown to downregulate activating NK cell receptors as well as the secretion of NK effector molecules such as perforin and granzyme B [54,55].

Contrary to primary NK cells, there is very few data investigating effects of the immunosuppressive TME for NK-92 cell-based immunotherapy. In the present study, we analyzed effectivity of CAR NK-92 cells targeting CD276 in vitro in the presence of low pH, hypoxia and other known factors of the TME.

## 2. Materials and Methods

### 2.1. Cell Lines and Culturing Conditions

Cutaneous melanoma cell lines FM-3 and WM115 were purchased from the European Collection of Authenticated Cell Cultures (ECACC, Wiltshire, UK), the cell line Mel-Juso was purchased from the German Collection of Microorganisms and Cell Cultures GmbH (DSMZ, Braunschweig, Germany), the cell lines M14, SK-MEL-2 and UACC62 were acquired from the American Type Cell Culture Collection (ATCC, Manassas, VA, USA) and the cell lines SK-MEL-5 and SK-MEL-28 were purchased from Cell Lines Services (CLS, Eppelheim, Germany). All tumor cell lines were maintained in RPMI 1640 GlutaMAX™ medium containing 2 mM stable l-glutamine and supplemented with 10% heat inactivated fetal bovine serum (FBS) (Thermo Fisher Scientific, Waltham, MA, USA), referred to as RPMI complete medium.

Dermal fibroblasts were purchase from PromoCell (Heidelberg, Germany) and cultivated in RPMI complete medium. Cancer-associated fibroblasts were established as a by-product from biopsy tumor samples provided by the Department of Orthopedic Surgery, University Hospital Tuebingen (UKT) and also cultivated in RPMI complete medium.

Lenti-X 293T cells (Takara Bio, Kyoto, Japan) were cultivated in DMEM high glucose (4.5 g/L) medium (Thermo Fisher Scientific, Waltham, MA, USA) containing GlutaMAX supplemented with 10% FBS and 1 mM sodium pyruvate (Thermo Fisher Scientific, Waltham, MA, USA). NK-92 cells were purchased from the American Type Culture Collection (ATCC) and maintained at a concentration of 10^5^ cells/mL in MEM Alpha Medium containing GlutaMAX (Thermo Fisher Scientific, Waltham, MA, USA) supplemented with 20% FBS and 100 U/mL IL-2 (Proleukin, Aldesleukin, Chiron, Emeryville, CA, USA) referred to as NK-92 complete medium.

All media contained 1× antibiotic-antimycotic solution (Thermo Fisher Scientific, Waltham, MA, USA) consisting of 100 U/mL of penicillin, 100 µg/mL of streptomycin and 0.25 µg/mL amphotericin B. All cells and cell lines were maintained at 37 °C in a humidified 5% CO_2_ atmosphere and were regularly tested for mycoplasma contamination.

### 2.2. Flow Cytometry

Cells were stained at 4 °C using the indicated antibodies in flow cytometry buffer made of PBS (Sigma-Aldrich, St. Louis, MO, USA) supplemented with 2% FBS and 0.5 M EDTA (Sigma-Aldrich, St. Louis, MO, USA) for 15 min. Using a BD FACSCanto II flow cytometer, live cells were gated based on forward and side scatter. CD276-CAR expression on NK-92 cells was determined by CD34 marker gene expression.

### 2.3. Generation of Lentiviral Vectors

A second generation lentiviral vector plasmid encoding the CD276-specific chimeric antigen receptor (CD276-CAR) construct was acquired from Creative Biolabs, Shirley, NY, USA. Exact constitution of the CD276-CAR construct was previously described [22]. The transfer plasmid encoding a GFP-luciferase construct was obtained from Dr. Irmela Jeremias, Helmholtz Center, Munich, Germany.

Lentiviral particles (LVP) were produced in Lenti-XTM 293T (Takara Bio, Kyoto, Japan) after lipofection (Lipofectamine 3000, Thermo Fisher Scientific, Waltham, MA, USA) of a second generation packaging plasmid, a VSV-G envelope plasmid and the indicated transfer plasmid. LVP containing supernatants were concentrated using Lenti-X concentrator (Takara Bio, Kyoto, Japan) and stored at −80 °C until further use.

### 2.4. Lentiviral Transduction

Exact transduction process of CD276-CAR-engineered NK-92 cells was described previously [22]. Melanoma cells were seeded at 5.0 × 10^4^ cells/mL or 1.25 × 10^5^ cells/mL in RPMI medium without supplements and subsequently transduced with GFP-luciferase LVP for 16 h. Cells were maintained in RPMI complete medium. Transduction efficiency was determined by flow cytometry.

### 2.5. Calcein-Release Cytotoxicity Assay (CRA)

Target cell staining with Calcein AM (Thermo Fisher Scientific, Waltham, MA, USA) as well as the protocol for the calcein release-based cytotoxicity assay (CRA) was described previously [22,56].

### 2.6. Real-Time Impedance-Based Live Cell Analysis

Melanoma cell lines were adjusted to a concentration of 10^5^ cells/mL in RPMI complete medium and seeded in E-Plate 96 VIEW (OLS, Bremen, Germany) micro-well plates. Effector CAR NK-92 cells were adjusted to an E:T ratio of 5:1 in NK-92 complete medium without IL-2 and co-incubated with the target cells. Utilizing the xCELLigence real-time cell analysis (RTCA) system, cells were monitored for at least 72 h. Melanoma cell viability was calculated using the RTCA 2.0 software and CAR mediated cytotoxicity was subsequently determined.

### 2.7. Cytokine Secretion Analysis

Cytokine release of CD276-CAR NK-92 cells was determined using the Bio-Plex Pro human cytokine 17-plex assay (Bio Rad, Hercules, CA, USA). The respective protocol was described previously [56].

### 2.8. Analysis of CD276-CAR NK-92 Cell-Mediated Cytotoxicity under Hypoxic Conditions

For the assessment of the influence of hypoxic conditions on cytotoxic efficacy of NK-92 cells, CD276-CAR-engineered as well as parental NK-92 cells were cultivated in a specialized incubator at 37 °C in a humidified 5% CO_2_, 1% O_2_ atmosphere for 24 or 168 h. Subsequently, they were utilized as effector cells in standard calcein release assays as described above under hypoxic conditions.

### 2.9. Testing the Influence of TGFβ on CD276-CAR NK-92 Cells

NK-92 and CD276-CAR NK-92 cells were cultivated in NK-92 complete medium with TGFβ at indicated concentrations for 48 h and subsequently used as effector cells in standard calcein release assays as described above.

### 2.10. Analysis of the Influence of Lactate on CD276-CAR NK-92 Cells

NK-92 and CD276-CAR NK-92 cells were cultivated in NK-92 complete medium with sodium l-lactate (Merck, Darmstadt, Germany) at indicated concentrations for 72 h. pH-values were measured using the LAQUAtwin pH-22 pH-meter (Horiba, Kyoto, Japan) and cells were subsequently used as effector cells in standard calcein release assays as previously described.

### 2.11. Generation of Melanoma Supernatants

FM-3, Mel-Juso and WM115 cells, human skin fibroblasts as well as cancer-associated fibroblasts (CAF) were seeded in 6-well plates in RPMI complete medium at a concentration of 6.25 × 10^4^ cells/mL. They were cultivated at 37 °C for 72 h. Cells were harvested, centrifuged and the medium supernatant was collected carefully. pH values were measured using the LAQUAtwin pH-22 pH-meter (Horiba, Kyoto, Japan).

Next, CD276-CAR NK-92 cells as well as parental NK-92 cells were cultivated in melanoma or fibroblast supernatants at a concentration of 10^5^ cells/mL for 48 h and subsequently used as effector cells in standard calcein release assays as described above.

### 2.12. Generation of Melanoma 3D Spheroids for the Assessment of CD276-CAR-Mediated Cytotoxicity of NK-92 Cells

GFP-transduced melanoma cells were adjusted to a concentration of 5.0 × 10^3^ cells/mL and seeded in a 96-well low attachment U-bottom plate (Nexcelom, Lawrence, MA, USA). Spheroids were grown for 72 h and were subsequently co-incubated with CD276-CAR NK-92 cells. Fluorescence was measured using the Celigo S imaging cytometer (Nexcelom, Lawrence, MA, USA) at indicated time points over a period of 96 h. CAR-mediated cytotoxicity was calculated using the average integrated fluorescence intensity of melanoma spheroids.

### 2.13. Tracking of NK-92-Mediated Tumor Cell Invasion

NK-92 as well as CD276-CAR NK-92 cells were assessed for their invasion potential. (CD276-CAR) NK-92 cells were stained using CellTracker™ Deep Red dye (Thermo Fisher Scientific, Waltham, MA, USA) according to the staining protocol provided by the manufacturer. Melanoma 3D spheroids were co-incubated with NK-92 cells at an E:T ratio of 5:1 and monitored using the Celigo S imaging cytometer (Nexcelom, Lawrence, MA, USA) at indicated time points over a period of 120 h.

### 2.14. Assessment of NK-92 Migration

For the assessment of NK-92 migration potential, GFP-transduced WM115 melanoma cells were grown as 3D spheroids according to protocol. Melanoma spheroids were then incubated with or without CXCL12 and/or prostaglandin E_2_ (PGE2) at indicated concentrations for the remaining experiment according to the publication by Berahovic at al. 2006 [57]. NK-92 cells as well as CD276-CAR NK-92 cells were seeded in a HTS Transwell-96 Well permeable supports with barcode, tissue culture treated, 5.0 µm pore size plate (Corning, Corning, NY, USA) at an E:T ratio of 20:1 in NK-92 complete medium without IL-2. The trans-well plate was put on top off the spheroid low-attachment plate to allow for NK-92 cell migration. After 24 h, the trans-well plate was removed and fluorescence of the spheroid was regularly measured using the Celigo S imaging cytometer (Nexcelom, Lawrence, MA, USA) over a period of 72 h. NK-92-mediated cytotoxicity was calculated using the average integrated fluorescence intensity of melanoma spheroids.

### 2.15. CRISPR/Cas9-Mediated Knock-Out of NKG2A in CD276-CAR NK-92 Cells

#### 2.15.1. SgRNA Design

Five different sgRNAs (Table 1) were employed to disrupt the NKG2A gene. NKG2A-1, NKG2A-2 and NKG2A-3 were designed using the CHOPCHOP software version 3 [58]. NKG2A-4 and NKG2A-5 sequences were obtained from the NKG2A Human Gene Knockout Kit (KN203062, OriGene, Rockville, MD, USA).

#### 2.15.2. Assessment of CRISPR/Cas9 In Vitro Cutting of NKG2A Gene

The cutting potential of the designed sgRNAs was determined by the in vitro cleavage of target DNA with ribonucleoprotein (RNP) complex protocol (IDT).

#### 2.15.3. Generation of CRISPR-Mediated Knock-Out of NKG2A Gene in CD276-CAR NK-92 Cells

V3 Cas9 RNP and chemically modified sgRNAs were incubated at a molar ratio of 1:2 (45 pmol to 90 pmol) at room temperature for 15 minutes [59]. After complex formation, 10^5^ NK-92 cells were transfected using the 10 µL Neon Transfection kit (ThermoFisher Scientific, Waltham, MA, USA) with the following electroporation settings: 1.200 V, 20 ms, 1 pulse (Neon System, ThermoFisher Scientific, Waltham, MA, USA). Finally, cells were transferred to a 48 well-plate in fresh NK-92 complete medium.

#### 2.15.4. Assessment of CRISPR-Mediated Modification of NKG2A at Genomic Level in CAR-NK-92 Cells

On day 5 post-electroporation, the CRISPR-modified cells were harvested for DNA isolation. DNA was extracted with the NucleoSpin Tissue kit following the manufacturer’s instructions (Macherey-Nagel, Düren, Germany) and was subsequently employed in a PCR reaction (100 ng). The genomic region including the CRISPR target site was specifically amplified with GoTaq Green DNA polymerase master mix (Promega, Madison, WI, USA) using following protocol: initial denaturation of 95 °C for 2 min followed by 40 cycles of 40 s at 95 °C, 40 s at 55 °C and 1 min at 68 °C. The PCR reaction was cleaned up from unincorporated nucleotides and other reagents with QIAquick PCR purification kit following the commercial protocol (Qiagen, Hilden, Germany) and then Sanger-sequenced (Table 2) by Eurofins Genomics (Konstanz, Germany). The results were further analyzed with ICE software (Synthego, Redwood City, CA, USA).

#### 2.15.5. Measurements of CRISPR-Mediated Knock-Out Score of NKG2A at Protein Level in NK-92 Cells

Cells were harvested for FACS analysis 5 days after electroporation. NKG2A-FITC antibody (130-114-091, Miltenyi Biotec, Bergisch Gladbach, Germany) was employed for flow cytometry analysis (FACS Calibur, BD Biosciences, Franklin Lakes, NJ, USA) following the commercial protocol. The gating strategy was set up using FlowJo software according to the positive population observed in the control sample. The knock-out score was calculated as the NKG2A negative population presented in the CRISPR-modified cells.

### 2.16. Data Analysis

All statistical analyses were performed with GraphPad Prism 8 software (GraphPad Software Inc., San Diego, CA, USA). Flow cytometry data were analyzed using FlowJo software V10.0.8 (FlowJo LLC., BD Biosciences, Franklin Lakes, NJ, USA).

## 3. Results

**Figure 1:**
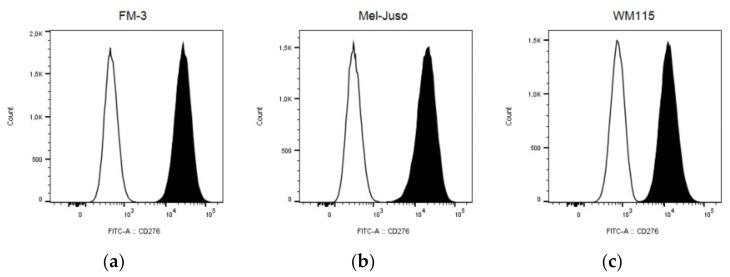
The commercially available melanoma cell lines FM‐3 (**a**), Mel‐Juso (**b**) and WM115 (**c**) were screened for their B7‐H3 (CD276) surface expression using flow cytometry. CD276 antigen expression (black) was determined using a FITC‐labeled anti‐CD276 antibody and compared to the respective isotype antibody (white).

B7-H3 was shown to be a promising option of targeted immunotherapy. We previously showed that CD276-CAR NK-92 cells were effective in targeting neuroblastoma cells in vitro [22]. To examine whether CD276-CAR NK-92 cells can also successfully target melanoma, commercially available melanoma cell lines, among others FM-3, Mel-Juso and WM115, were screened for their CD276 surface expression using flow cytometry (Figure 1).

All melanoma cell lines that were screened during this study showed uniform abundance of CD276 expression. As a proof of principle, the three cell lines FM-3, Mel-Juso and WM115 were chosen for subsequent experiments.

We characterized the cell lines for surface expression of important NK cell ligands using flow cytometry (Table 3). All cell lines showed abundant expression of various types of human leukocyte antigens, including HLA-E, a major ligand for the inhibitory receptor complex CD94/NKG2A on NK cells. Furthermore, high expression levels of the major histocompatibility complex (MHC) class I chain-related protein A and B (MICA/B), a protein that, upon binding to the natural killer group 2, member D receptor NKG2D (CD314), induces an activating signaling cascade in NK cells, could be demonstrated for the FM-3 and Mel-Juso cell lines. Moreover, all three cell lines expressed Nectin 2 (CD112), PVR (CD155) as well as death receptor 5 (CD262). By binding to the tumor necrosis factor (TNF)-related apoptosis-inducing ligand (TRAIL), CD262 can induce NK-mediated apoptosis. CD112 and CD155 are pivotal ligands for the DNAX Accessory Molecule-1 DNAM-1 (CD226) triggering NK cell activation. However, CD112 as well as CD155 can also induce an inhibitory cascade by activation of the immune checkpoint receptor TIGIT on NK cells.

**Figure 2:**
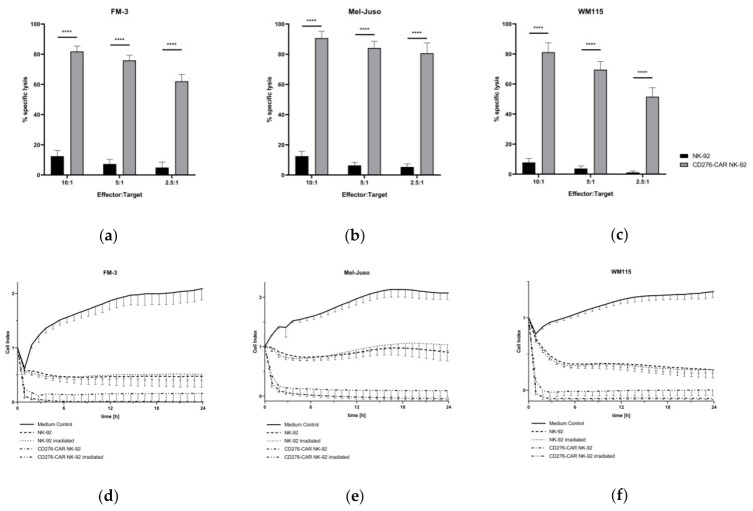

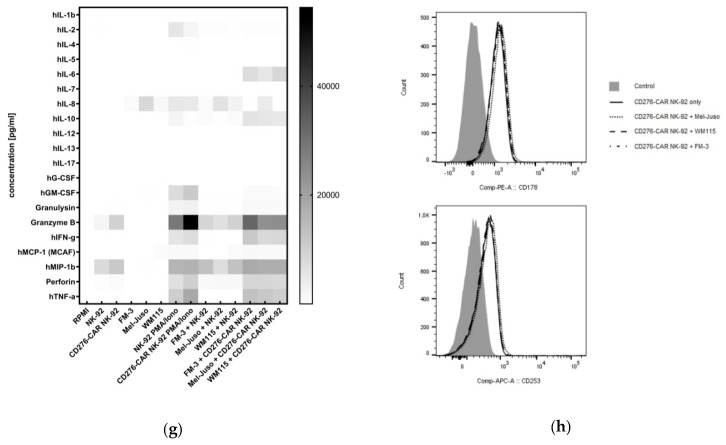
CD276-CAR NK-92 cells were co-incubated with calcein-labeled melanoma cell lines at indicated effector-to-target (E:T) ratios. Specific cytotoxicity was measured after 2 h and is shown as mean ± SD, *n* = 3 (**a**–**c**). To assess the kinetics of CD276-CAR-mediated tumor lysis, irradiated and non-irradiated CD276-CAR NK-92 as well as parental NK-92 cells were co-incubated with unlabeled melanoma cells at an E:T ratio of 5:1. Tumor cell viability was monitored using the real-time live cell analysis (RTCA) xCELLigence system, *n* = 3 (**d**–**f**). NK-92 cytokine secretion in the presence or absence of melanoma target cells was determined using the Bio-Plex Pro human cytokine 17-plex assay and is shown as a heatmap (**g**). CD276-CAR NK-92 cell surface expression levels of FasL (CD178) and TRAIL (CD253) were screened using flow cytometry before and after co-incubation with melanoma cell lines (**h**); **** *p* < 0.0001.

Next, the melanoma cell lines were used as target cells in standard calcein release assays (CRA) to assess the specific cytotoxic potential of CD276-CAR NK-92 cells (Figure 2a–c). CD276-CAR NK-92 cells as well as parental NK-92 cells were co-incubated with calcein-labeled melanoma cells. After two hours, a significant increase in specific CAR-mediated tumor lysis could be demonstrated for all three cell lines. Even at low E:T ratios, specific cytotoxicity increased up to 70%. Previously, we have shown that CD276-CAR NK-92 cells showed no undesirable off-target effects since CAR-mediated cytotoxicity only occurred in the presence of CD276 surface expression on target tumor cells [22]. In order to examine the kinetics of CAR-mediated cytolysis, CD276-CAR NK-92 as well as untransduced NK-92 cells were co-incubated with unlabeled melanoma cells and monitored using the xCELLigence real-time cell analysis (RTCA) system (Figure 2d–f). Electrical impedance is measured and interpreted as the dimensionless cell index. Increase in cell index describes tumor cell growth while cell index decrease is proportional to tumor cell death. A significant decrease in tumor cell viability could be shown within two hours upon addition of CD276-CAR NK-92 cells and no tumor regrowth was detectable over a longer period of time. Furthermore, we tested whether γ-irradiation of NK-92 cells with 10 Gy, as required in all NK-92 clinical trials, would decrease CAR-mediated cytotoxic potential. We found that the difference between irradiated and non-irradiated cytotoxicity was only marginal and did not reach statistical significance.

Next, a cytokine secretion profile for CD276-CAR and parental NK-92 cells upon co-incubation with melanoma cells was established to screen for the release of NK effector molecules (Figure 2g). Compared to parental NK-92 cells, co-incubation of CD276-CAR NK-92 cells with melanoma cells significantly increased secretion of various interleukins, among others, IL-6 (98- to 317-fold) and IL-10 (6- to 15-fold). Furthermore, a drastic increase of pro-inflammatory cytokines such as IFN-γ (44- to 81-fold) and TNF-α (101- to 225-fold) could be detected. Increase of NK effector molecule release, granulysin (4-fold), granzyme B (3- to 4-fold) and perforin (5- to 7-fold), was evenly distributed. Additionally, flow cytometric analysis showed no increase in surface expression of two important pathways in NK-mediated apoptosis induction, Fas ligand (FasL) and TNF-related apoptosis-inducing ligand (TRAIL), in CD276-CAR NK-92 cells upon co-incubation with melanoma cells (Figure 2h).

The immunosuppressive microenvironment surrounding solid tumors has been shown to negatively affect cytotoxic potential of NK cells. We examined how hypoxic or acidic culture conditions and soluble factors secreted by cancer cells or cancer-associated, such as transforming growth factor beta (TGFβ), influence CAR-mediated effector function of NK-92 cells.

**Figure 3:**
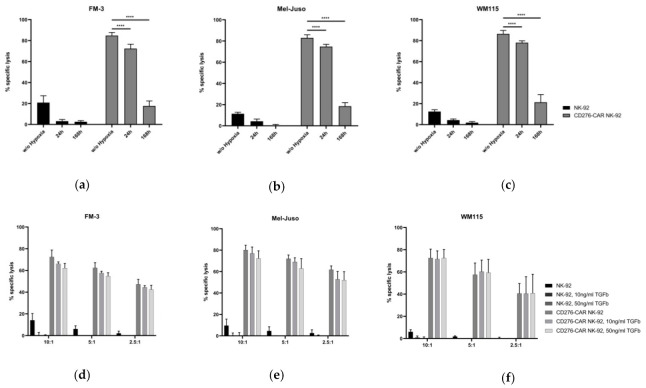

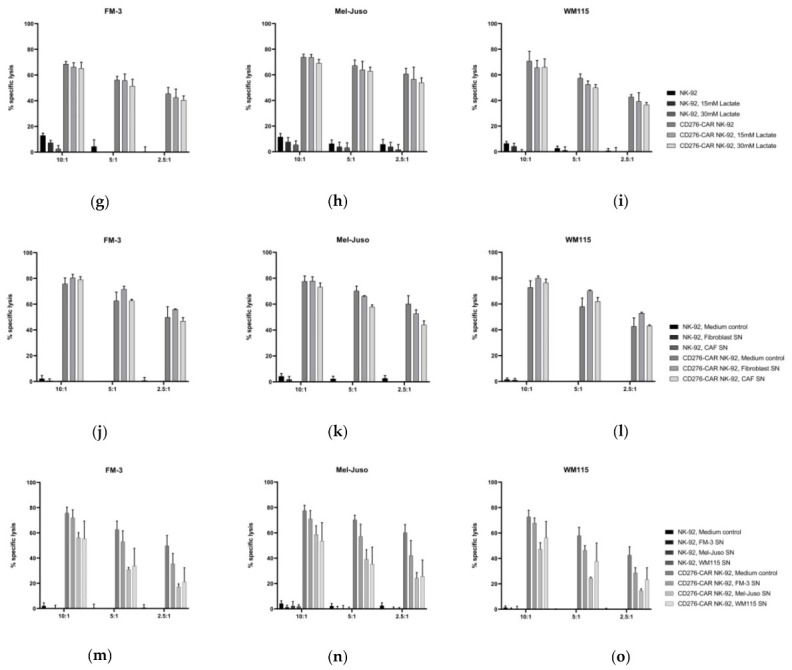
CD276-CAR NK-92 cells as well as parental NK-92 cells were co-incubated with calcein-labeled melanoma cell lines at indicated E:T ratios for 2 h. Specific cytotoxicity was assessed using a calcein release assay and is shown as mean ± SD, *n* = 3. Prior to the experiments, NK-92 and CD276-CAR NK-92 cells were either cultivated under hypoxic conditions (5% CO_2_, 1% O_2_) for 24 and 168 h (**a**–**c**), pre-treated with indicated concentrations of TGFβ (**d**–**f**), sodium l-lactate (**g**–**i**) or supernatants, previously collected from human fibroblast and cancer-associated fibroblast (CAF) culture (**j**–**l**) as well as melanoma cell line culture (**m**–**o**), for 48 h; **** *p* < 0.0001.

First, NK-92 and CAR-transduced NK-92 cells were cultivated under hypoxic conditions (5% CO_2_, 1% O_2_) for 24 and 168 h. In a standard CRA, compared to cells cultivated in standard conditions, CD276-CAR NK-92 cells showed slightly decreased cytotoxic potential after hypoxic cultivation for 24 h but after cultivation for 168 h, specific CAR-mediated cytotoxicity drastically decreased to circa 20% (Figure 3a–c). Importantly, NK-92 cell viability was also impaired by 30% and 80%, respectively. Next, we investigated the effect of the immunosuppressive TGFβ. CD276-CAR NK-92 and NK-92 cells were cultivated with soluble TGFβ at concentrations of 10 ng/mL and 50 ng/mL for 48 h. Cytotoxic efficacy was assessed in a CRA and compared to untreated NK-92 and CD276-CAR NK-92 cells (Figure 3d–f). We found no significant decrease in cytotoxicity against all three melanoma cell lines at any E:T ratio, which suggests that TGFβ seems to have no negative effect on CD276-CAR NK-92-mediated tumor lysis.

Acidosis, as a fundamental feature of the TME, can impact functionality and tumor cell invasion capacity of primary immune cells such as NK cells. Here, we assessed whether an acidic environment during cell culture affects NK-mediated cytotoxicity of NK-92 cells. CD276-CAR and parental NK-92 cells were cultivated in NK-92 complete medium with different concentrations of sodium l-lactate for 48 h (Figure 3g–i). Cell supernatant pH values decreased from 8.0 in an untreated control to 7.5 and 7.2 in medium treated with 15 or 30 mM sodium l-lactate, respectively. Cytotoxic effector function of NK-92 as well as CAR-transduced NK-92 cells was not significantly impaired by acidity of culture medium. Notably, NK-92 cell proliferation and viability were not impaired either.

Lastly, we wanted to determine if incubation of NK-92 cells in the presence of soluble factors secreted by melanoma cells, such as TGFβ, IL-10, IDO, nitric oxide (NO) and prostaglandins, molecules that have been shown to be regularly secreted by tumor cells and to induce an immunosuppressive phenotype in primary immune cells, could also weaken immune effector function of NK-92 cells [60,61,62,63]. The melanoma cell lines FM-3, Mel-Juso and WM115 as well as human fibroblasts and cancer-associated fibroblasts were cultivated for 72 h. Supernatant was harvested and pH value was assessed. Compared to the control medium, pH values in the supernatant decreased from 8.0 to 7.4 ± 0.1. CD276-CAR and NK-92 cells were subsequently incubated in tumor cell supernatant for 48 h and used as effector cells in a CRA with all three melanoma cell lines (Figure 3j–o). Incubation of CD276-CAR NK-92 cells in supernatant from FM-3 culture did not impact cytotoxicity while CAR NK-92 cells incubated with Mel-Juso as well as WM115 cell culture supernatant did show slightly decreased CAR-mediated cytolysis of all three melanoma cell lines. Interestingly, cultivation in medium collected from fibroblasts and CAF did not impact CAR-mediated NK effector function.

All in all, no negative effect on CD276-CAR NK-92 cell-mediated cytotoxicity could be demonstrated with variances in acidity, immunosuppressive molecules like TGFβ or co-incubation with cancer-associated fibroblasts. Only long-lasting hypoxic culture conditions as well as incubation with tumor cell supernatants slightly affected CD276-CAR NK-92 cell function.

**Figure 4:**
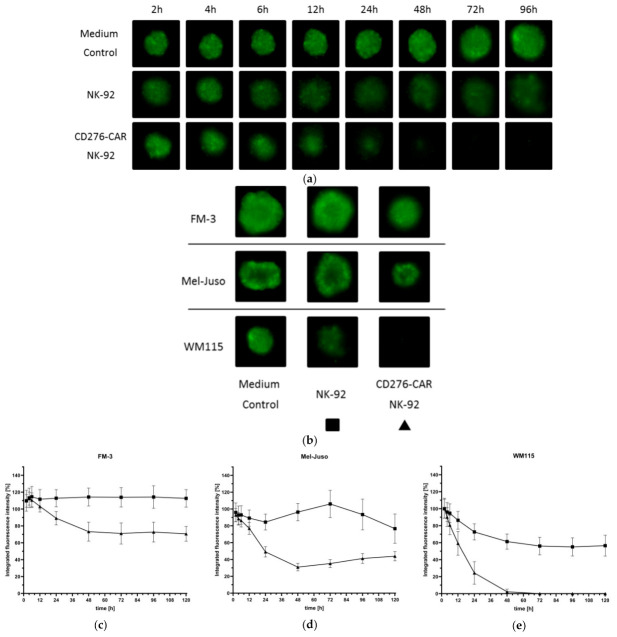
GFP-transduced cell lines FM-3, Mel-Juso and WM115 were grown as three-dimensional melanoma spheroids for 72 h before being co-incubated with CD276-CAR NK-92 or parental NK-92 cells for 96 h. Integrated fluorescence intensity of melanoma spheroids (FM-3, Mel-Juso and WM115) was measured regularly using the Celigo S imaging cytometer. Representative fluorescence images of WM115 spheroids from indicated time points (**a**) as well as all melanoma spheroids after 96 h (**b**) are shown. Integrated fluorescence intensity was compared to untreated control spheroids and is shown as mean ± SD, *n* = 3 (**c**–**e**).

Evaluation of immune effector function in in vitro assays is usually restricted to two-dimensional tumor cell culture. The 3D cell cultures, better mimicking the in vivo situation, are physiologically more relevant and predictive than standard 2D assays. Therefore, three-dimensional melanoma spheroid models were established to examine whether CD276-CAR NK-92 prove to be as effective in tumor cell lysis as in a standard CRA. Melanoma cell lines were grown as spheroids for 72 h according to the protocol published by Vinci et al. 2012 and subsequently co-incubated with CD276-CAR NK-92 cells. Representative fluorescence pictures of tumor spheroids were taken regularly (Figure 4a–b) and compared to co-incubation with untransduced NK-92 cells. CAR-mediated tumor cell lysis of each of the three tumor cell lines was drastically increased after no later than 24 h after addition of NK effector cells (Figure 4c–e). Notably, WM115 spheroids were lysed fully after 48 h and tumor cell regrowth could not be observed at a later time point.

**Figure 5:**
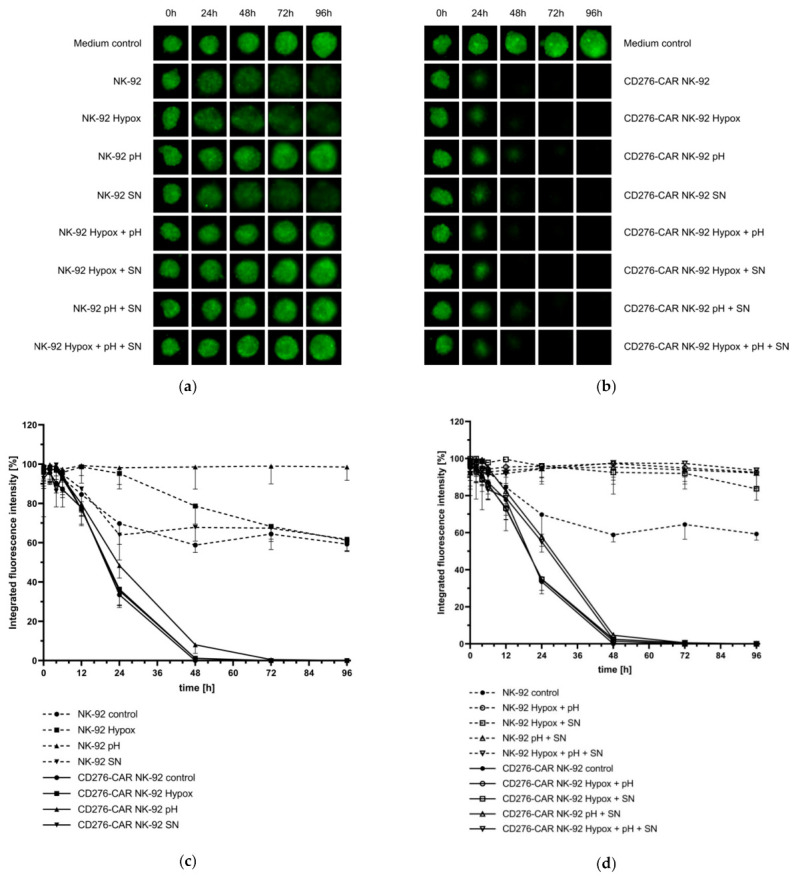
The GFP-transduced cell line WM115 was grown as 3D spheroid for 72 h. CD276-CAR NK-92 and parental NK-92 cells had been cultivated under hypoxic conditions, under low pH, with WM115 cell culture SN or various combinations thereof for 48 h and were added to the melanoma spheroids. Integrated fluorescence intensity of spheroids was measured regularly over 96 h using the Celigo S imaging cytometer. Representative fluorescence images of WM115 spheroids co-incubated with NK-92 (**a**) and CD276-CAR NK-92 (**b**) from indicated time points are shown. Average integrated fluorescence intensity was compared to untreated control spheroids and is shown as mean ± SD, *n* = 3 (**c**–**d**).

We then utilized melanoma spheroids to assess the potential impairment of CAR-mediated cytotoxicity by the TME factors, thus, mimicking a more realistic in vivo scenario. Therefore, CD276-CAR NK-92 and parental NK-92 cells were cultivated under hypoxic conditions, under low pH, with WM115 cell culture SN and various combinations thereof for 48 h and then co-incubated with WM115 spheroids for 96 h. Fluorescence images of the melanoma spheroids were taken regularly (Figure 5a–b). Integrated fluorescence intensity of spheroids co-incubated with NK-92 and CD276-CAR NK-92 cells were compared to untreated control spheroids (Figure 5c–d). All melanoma spheroids that were co-incubated with CD276-CAR NK-92 cells were completely eradicated after 48 to 72 h. Only pre-cultivation of CAR NK-92 cells in medium with lactate-induced low pH slightly decreased the time course of tumor cell lysis compared to untreated cells. However, it did not impair overall cytotoxic function. Interestingly, parental NK-92 cells that had prior been cultivated in low pH showed significantly lower cytolytic effects compared to untreated NK-92 cells.

Overall, CAR-mediated cytotoxicity of melanoma cells did not drastically decrease after incubation of effector cells with various combinations of the immunosuppressive factors of the TME in either 2D or 3D cell culture systems.

**Figure 6:**
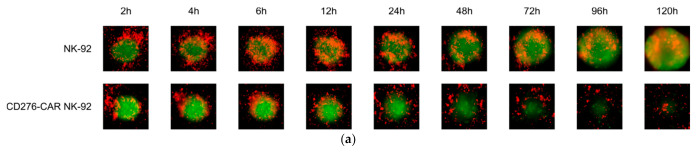

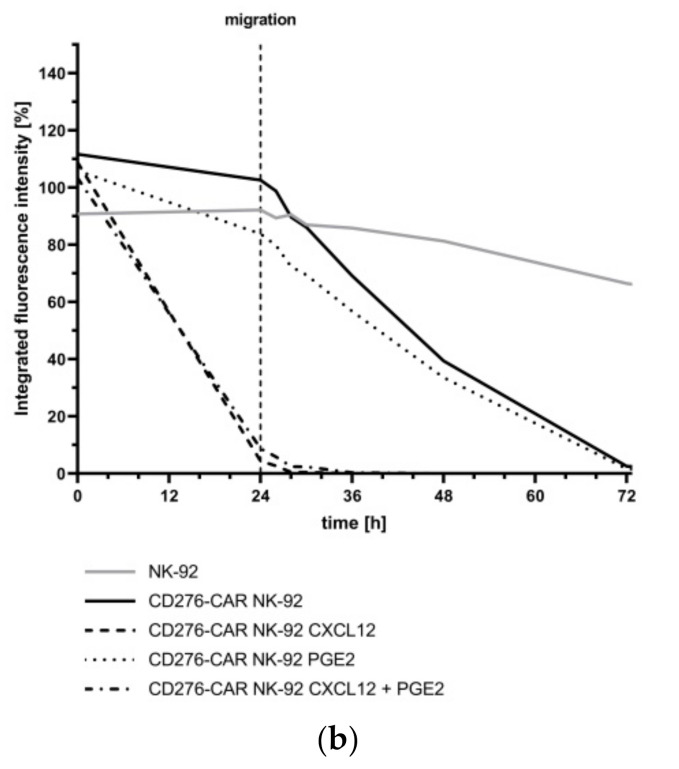
GFP-transduced WM115 melanoma cells were grown as 3D spheroids and co-incubated with CellTracker™ Deep Red-labeled NK-92 or CD276-CAR NK-92 cells for 120 h. Representative fluorescence pictures of spheroids at indicated time points are shown (**a**). WM115 spheroids were incubated with or without CXCL12 at a concentration of 100 nM. NK-92 cells (grey line) as well as CD276-CAR NK-92 cells (black lines) were pre-treated with or without prostaglandin E2 (PGE2) at a concentration of 5.7 µM and seeded in a HTS trans-well plate at an E:T ratio of 20:1. Cells were allowed to migrate for 24 h. Spheroid fluorescence was regularly measured using the Celigo S imaging cytometer over a period of 72 h. NK-92-mediated cytotoxicity was calculated using the average integrated fluorescence intensity of melanoma spheroids and is shown as mean (**b**); *n* ≥ 3.

For robust immunotherapy of solid tumors, primary immune cells need to be able to deeply infiltrate tumor tissue. To assess NK-92 tumor infiltration potential, NK-92 as well as CD276-CAR NK-92 cells were labeled with a red fluorescent dye and co-incubated with GFP-transduced WM115 spheroids for 120 h. Fluorescent images of the cells were taken regularly at indicated time points (Figure 6a). Interestingly, parental NK-92 cells seem to infiltrate tumor spheroids faster than their CAR-transduced counterparts but without increased tumor cell lysis. On the contrary, CD276-CAR NK-92 cells show high cytotoxic potential lysing tumor spheroids from the outside, indicating that lysis of the spheroids is not dependent on complete infiltration.

Migration potential is another important factor for the treatment efficacy of solid tumors using immune cells. Therefore, NK-92 migration potential was assessed using permeable trans-well plates. GFP-transduced WM115 tumor spheroids were grown according to protocol and CD276-CAR NK-92 cells were subsequently added in tissue culture-treated wells with 5.0 µm pore size. Untransduced NK-92 cells were used as controls. A small sample of tumor culture medium was supplemented with soluble CXCL12 at a concentration of 100 nM to enhance immune cell migration and some CD276-CAR NK-92 cells were pre-treated with prostaglandin E2 (PGE2) at a concentration of 5.7 μM in order to block migration [64,65]. CXCL12 is a highly effective chemoattractant that is expressed in various cell types, including melanoma [66]. It binds to, among others, the C-X-C chemokine receptor type 4 (CXCR-4), which is expressed in NK-92 cells [22]. Cells were allowed to migrate for 24 h and integrated fluorescence intensity of tumor spheroids was measured regularly (Figure 6b). CD276-CAR NK-92 cells that were added to tumor spheroids in the presence of CXCL12 were able to migrate quickly and show high cytotoxic potential within 24 h. In the absence of supplemented CXCL12, cells seem to migrate in lower numbers, so a delayed tumor lysis was observed. Notably, the pre-treatment of NK-92 cells with PGE2 did not impair migration or cytotoxic potential independent of CXCL12 supplementation of tumor medium. To sum up, CD276-CAR NK-92 cells demonstrated the ability to effectively migrate to tumor sites and infiltrate and lyse melanoma cells in a 3D spheroid model system.

In the context of TME-mediated immune escape strategies, HLA-E expression is often elevated in melanoma [67]. We found abundant HLA-E expression in all melanoma cell lines that were used in this study. Its respective NK receptor, NKG2A (CD159a), is one of the few inhibitory receptors expressed by NK-92 cells. In order to assess the influence of HLA-E binding to NKG2A for NK-92-mediated cytotoxicity and to potentially further enhance the efficacy of the generated CD276-CAR NK-92 cells, the disruption of NKG2A (CD159a) was studied.

**Figure 7:**
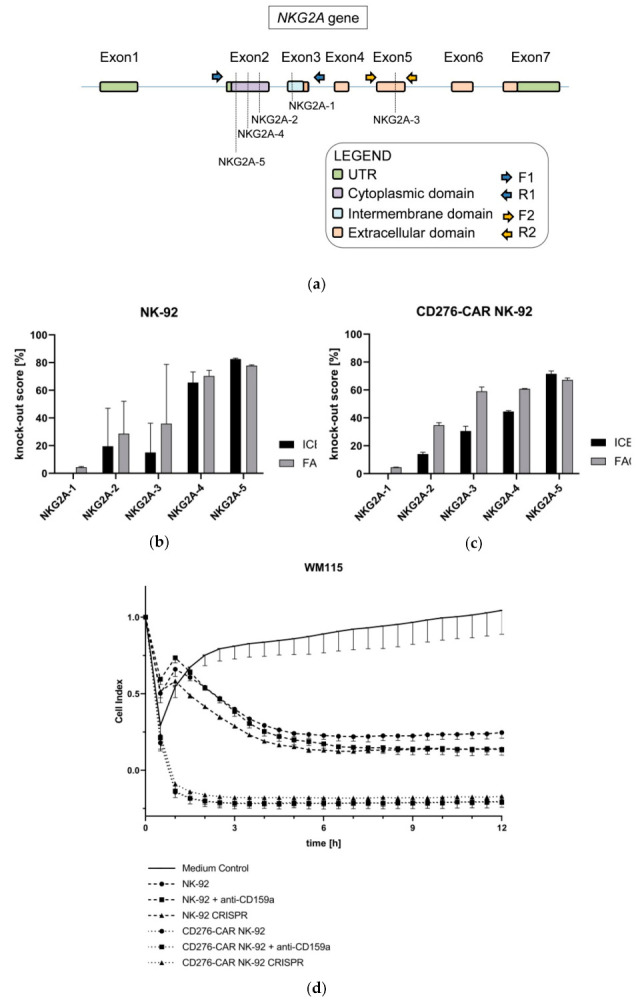
The schematic illustration shows the NKG2A (CD159a) gene and five potential sgRNA cutting sites in exons 2, 3 and 5 (**a**). Knock-out scores for NK-92 and CD276-CAR NK-92 cell NKG2A knock-out were calculated using Sanger sequencing (black) and flow cytometric analysis (grey) (**b**,**c**). Cytotoxic potential of knock-out variant 5 (NKG2A-5) was assessed using the xCELLigence real-time cytotoxicity assay and compared to unmodified controls as well as wildtype CD276-CAR and parental NK-92 cells that had been pre-treated with an inhibitory anti-NKG2A antibody (**d**); *n* = 3.

The predominance of NKG2A^+^ NK cells in the tumor microenvironment reveals its importance in the suppressive axis between tumor and NK cells [68], leading to important clinical studies using anti-NKG2A inhibitory antibodies in the treatment of cancer [69,70]. As a matter of fact, the disruption of NKG2A in NK cells was proven to overcome tumor resistance [71]. Therefore, we hypothesized that the disruption of *KLRC1* (encoding for NKG2A protein) would boost the antitumor activity of CD276-CAR NK-92 as a result of bypassing this immune scape mechanism. To corroborate this idea, five different sgRNAs aiming for the coding sequence of the gene were considered. Three of them (NKG2A-2, NKG2A-4 and NKG2A-5) target the cytoplasmic domain, one (NKG2A-1) the intermembrane domain and one (NKG2A-3) the intracellular domain (Figure 7a). The obtained results revealed that NKG2A-5 induced the highest percentage of indels (82.5% and 71.5% in NK-92 and CD276-CAR NK-92 cells, respectively), and so achieved the highest level of disruption (77.8% and 67.2%) at protein level (Figure 7b–c).

The knock-out cells obtained by NKG2A-5 editing were subsequently sorted and expanded to generate pure NKG2A-KO NK-92 and CD276-CAR NK-92 cell lines. Next, the cells were co-cultured with the different melanoma cell lines in standard CRA. Slightly increased tumor cell lysis of melanoma cells was observed for NKG2A-KO NK-92 cells in comparison to their unmodified counterparts. However, this improvement was not demonstrated in NKG2A-KO CD276-CAR NK-92 cells, where the reported cytotoxic performance was similar to unmodified CD276-CAR NK-92 cells (Figure 7d). This was further underscored by pre-treatment of CD276-CAR NK-92 cells with a blocking antibody targeting NKG2A which, similarly, did not enhance CAR-mediated cytotoxicity suggesting that cytotoxic potential of CD276-CAR NK-92 cells is either not influenced by inhibitory ligands or the CAR-induced activation signal is strong enough to override inhibitory signaling.

## 4. Discussion

Melanoma is one of the most common cancers worldwide with its incidence continuing to rise each year [72]. Even though immune checkpoint blockade drastically increased overall outcome for some patients, new treatment strategies for therapy-resistant melanomas are in dire need.

Chimeric antigen receptor-engineered T cells provide a therapeutic approach that involves the use of modified primary lymphocytes to specifically target cancer cells. Currently, only seven clinical trials examining the usage of CAR T cells for the treatment of melanoma, targeting among others disialoganglioside GD2 or vascular endothelial growth factor receptor 2 (VEGFR2), are on-going, while no clinical trials have yet been conducted with other CAR-engineered immune cells such as primary NK cells. Major obstacles for CAR efficiency in solid tumors include the identification of tumor-specific antigens as well as tumor antigen heterogeneity [24]. Additionally, due to the presence of the highly immunosuppressive microenvironment in this context, clinical results so far have been underwhelming [73].

One very promising clinical study, conducted at the Seattle Children’s Hospital, is targeting the immune checkpoint molecule CD276 [74]. CD276 recently emerged as an important prognostic marker for various solid tumor entities and, thus, seems to be a promising immunotherapeutic target structure [25,26]. It was already shown in multiple studies that CD276 is aberrantly expressed in malign melanoma and the present study demonstrated CD276 abundance in all examined melanoma cell lines [75,76,77].

However, CAR T and CAR NK cells, regardless of their specific target structure, face complex obstacles when used in solid tumors. They need to successfully migrate through tissue to reach the tumor site, efficiently invade the tumor and also survive the suppressive TME that decreases anti-tumor response using various factors [78,79,80]. In the present study, we evaluated whether the recently established NK cell line CD276-CAR NK-92, that proved to be effective in targeting neuroblastoma, could also be an effective cellular product for the treatment of melanoma [22]. Furthermore, it was evaluated whether CAR-modified NK-92 cells are able to overcome negative factors of the TME, thus, making NK-92 cells a superior CAR vehicle for the treatment of melanoma and solid tumors in general.

Dynamic alterations during melanoma progression include the increase of growth factor secretion and, subsequently, growth of additional fibrous tissue surrounding the tumor site which in turn impairs effector cell migration [81]. Here, we showed that CD276-CAR NK-92 cells demonstrated successful eradication of various melanoma cell lines in vitro in 2D as well as 3D experiments. They were able to successfully migrate through small pores to the tumor site and penetrate melanoma spheroids while retaining their cytotoxic efficacy.

After successful extravasation, CAR-modified immune cells need to survive the unfavorable TME. The predominantly hypoxic and acidic conditions in the TME in combination with an anti-inflammatory cytokine milieu consisting of, among others, TGFβ can significantly impair CAR-mediated effector function [82,83,84,85,86]. TGFβ was demonstrated to directly impair NK cell functionality by downregulating activating NK receptors such as NKG2D and NKp30 or by suppressing the mTOR pathway [48,87]. Contrary to primary NK cells, CD276-CAR NK-92 cells showed significant resistance to soluble immunomodulatory factors. The cells retained their cytotoxic potential even after prolonged co-incubation with high concentrations of TGFβ.

Acidosis in the TME, caused by tumor cell secretion of substantial amounts of lactate, is associated with impaired cytotoxicity of primary NK cells as well as downregulation of activating NK cell receptors [88,89]. Conversely, effector function of CD276-CAR NK-92 cells was not impaired after cultivation with high concentrations of supplemented lactate. Moreover, cultivation in cell culture supernatant that was harvested from fibroblasts and cancer-associated fibroblasts, that play a pivotal role in tumor neo-vascularization, had also no detectable impact on CAR-mediated tumor lysis while melanoma supernatant only slightly affected CD276-CAR NK-92 cell function [90,91].

Hypoxic culture conditions for longer periods of time were drastically decreasing CAR-mediated target cell lysis of primary NK cells and NK-92 cells [54,55,92]. Since NK-92 cells have to be irradiated for clinical use to prevent uncontrolled in vivo proliferation due to being derived from a non-Hodgkin lymphoma, cell viability and effective cytotoxicity is restricted to a 48–72 h time window after irradiation. We previously demonstrated that application of CD276-CAR NK-92 cells immediately after irradiation did not impair CAR-mediated cytotoxicity [22]. Therefore, decreased immune effector function induced by long-term exposure to hypoxia (>72 h) is not a critical issue.

NKG2A is, contrary to primary NK cells, one of the few inhibitory receptors expressed by CD276-CAR NK-92 cells and their respective tumor cell ligands, HLA-E and HLA-G, are commonly overexpressed in cutaneous melanoma [22,56,71]. Furthermore, HLA-E and HLA-G expression levels are often elevated in the course of typical melanoma immune escape strategies [67]. A CRISPR-mediated NKG2A knock-out as well as blocking the NKG2A receptor with an inhibitory antibody did not significantly boost CAR-mediated cytotoxic potential of CD276-CAR NK-92 cells, suggesting that CAR effectivity in CD276-CAR NK-92 cells is independent of inhibitory ligands. Since the CAR-mediated cytotoxicity exerted by parental CD276-CAR NK-92 itself is already very strong, we hypothesize that the implementation of the described CRISPR protocol for other NK-92-based immunotherapies could improve the outcome of the respective treatment.

The accumulated data for CD276-CAR NK-92 cells and their resistance to the tumor microenvironment is very promising. Other factors, including the influence of immunosuppressive cell such as myeloid-derived suppressor cells (MDSC) or CAR NK-92 cell trafficking to the tumor site, need to be investigated further. In the near future, we are planning on establishing various in vivo models, including PDX and humanized mouse models, to validate these preliminary results.

Combining the aforementioned positive features of a CD276-CAR, targeting a tumor-specific antigen, with the NK-92 cell line and its strong resilience to influences exerted by the TME generates a powerful cellular product that can be easily manufactured under GMP guidelines as a readily available, “off-the-shelf” treatment option for melanoma and other solid tumors.

## Figures and Tables

**Table 1:** Nucleotide sequences for NKG2A sgRNAs

Name	Nucleotide Sequence
NKG2A-1	GAAGCTCATTGTTGGGATCC
NKG2A-2	TTGAAGGTTTAATTCCGCAT
NKG2A-3	ACTGGAGTTCTTCGAAGTAC
NKG2A-4	AGGCAGCAACGAAAACCTAA
NKG2A-5	GGTCTGAGTAGATTACTCCT

**Table 2:** Nucleotide sequences for NKG2A PCR primers

Primer	Nucleotide Sequence
Forward 1	TACTCGTTCTCCACCTCACC
Reverse 1	TAACGTGAAAATTCCCCTTGTAATC
Forward 2	ATTTACCAGCCCATGAAGATGT
Reverse 2	TCCATGAAAAGCAAAAACTGAA

**Table 3:** Characterization of melanoma cell lines for NK ligand expression. Tumor cells were co-incubated with fluorescently labeled antibodies and analyzed using flow cytometry. MFI values were calculated using staining with the respective isotype control antibody.

	CD48	CD50	CD54	CD58	CD95	CD102	CD112	CD155	CD261	CD262
**FM-3**	1.50	1.49	22.71	34.67	3.08	1.28	9.17	56.28	0.97	9.65
**Mel-Juso**	1.38	1.48	9.40	55.23	0.93	1.26	15.70	80.10	2.79	6.23
**WM115**	1.49	1.65	1.98	144.49	3.45	1.21	5.46	22.80	1.63	9.40
	**HLA-ABC**	**HLA-DR**	**HLA-E**	**HLA-G**	**MICA/B**	**ULBP1**	**ULBP2/5/6**	**ULBP3**	**ULBP4**	
**FM-3**	34.24	14.53	4.89	20.68	11.37	0.49	2.52	0.48	1.20	
**Mel-Juso**	30.86	33.69	7.11	22.73	21.45	0.58	7.03	0.78	0.20	
**WM115**	87.21	44.80	8.07	21.88	3.12	0.66	2.43	0.52	0.42	

## Data Availability

The data presented in this study are available from the corresponding author upon reasonable request.
